# Detection of a Chirality-Induced Spin Selective Quantum Capacitance in α-Helical Peptides

**DOI:** 10.1021/acs.nanolett.3c02483

**Published:** 2023-08-31

**Authors:** Pius Markus Theiler, Christian Ritz, Raphael Hofmann, Andreas Stemmer

**Affiliations:** † Nanotechnology Group, ETH Zürich, Säumerstrasse 4, 8803 Rüschlikon, Switzerland; ‡ Laboratory of Organic Chemistry, Department of Chemistry and Applied Biosciences, ETH Zürich, 8093 Zürich, Switzerland

**Keywords:** chirality-induced spin selectivity, Kelvin probe force microscopy, quantum capacitance, spinterface

## Abstract

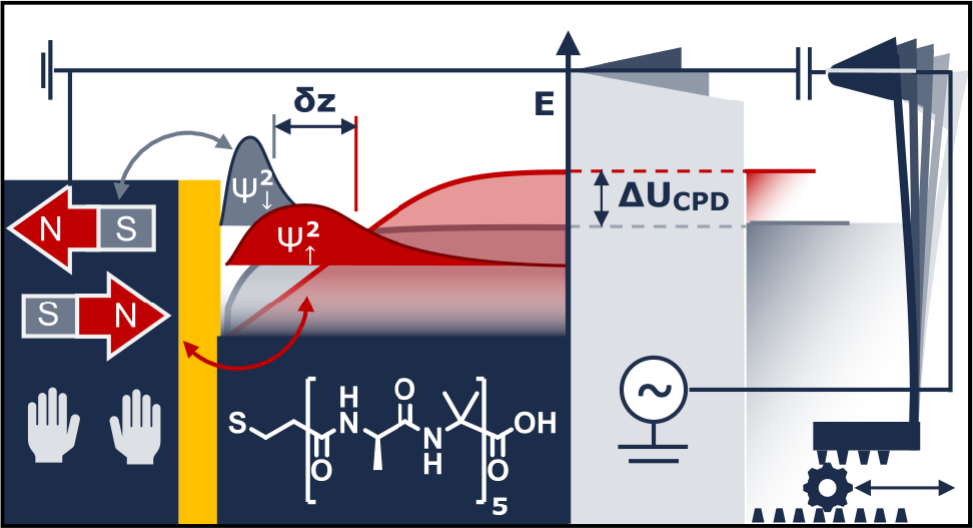

Advanced Kelvin probe force microscopy simultaneously detects the quantum capacitance and surface potential of an α-helical peptide monolayer. These indicators shift when either the magnetic polarization or the enantiomer is toggled. A model based on a triangular quantum well in thermal and chemical equilibrium and electron–electron interactions allows for calculating the electrical potential profile from the measured data. The combination of the model and the measurements shows that no global charge transport is required to produce effects attributed to the chirality-induced spin selectivity effect. These experimental findings support the theoretical model of Fransson et al. *Nano Letters***2021**, *21* (7), 3026–3032. Measurements of the quantum capacitance represent a new way to test and refine theoretical models used to explain strong spin polarization due to chirality-induced spin selectivity.

Chiral molecular materials show a high degree of spin polarization even at room temperature despite their low mass constituting elements and weak atomic spin–orbit coupling. The chirality of the material and the charge transport direction determine the preferential spin orientation. The effect is known as the chirality-induced spin selectivity (CISS) effect.^1^

There has yet to be a consensus on how to explain the mechanistic origins of the effect.^1^ One challenge is that theoretical predictions are difficult to test because of the need for experimental access to (multiple) direct observables.^2^ The effect is demonstrated in many different setups such as photoemission,^3,4^ conductive atomic force microscopy (AFM),^4−6^ scanning tunneling microscopy,^7,8^ and cyclic voltammetry.^4^ In all of these experiments, the charge is transported through the chiral material. It may lead to the hasty conclusion that nonequilibrium transport induces CISS.

However, the spinterface^9^ provides a route to get spin polarization without transport. For example, in molecular adsorption experiments on magnetic interfaces, there is no net steady-state current observed, and yet persistent effects on the adsorption density^10−12^ or magnetization^12−14^ are observed. Preliminary Hall-bar^15^ and magneto-optic Kerr effect^16^ measurements of peptide monolayer on thin gold films point in a similar direction, showing magnetization independent of externally applied fields. Ghosh et al.^11^ used Kelvin probe force microscopy^17^ (KPFM) to detect CISS-related changes in contact potential difference upon reversal of the magnetic polarization of the substrate or flipping the enantiomer. Further, experimentally measured spin accumulations at interfaces raise speculations about whether CISS involves chirality which changes handedness under time-reversal operation.^18^

Figure 1 shows a replication of the experiment of Ghosh et al.^11^ with frequency-modulated (FM) KPFM.^19^ KPFM is an atomic force microscopy technique to quantify the electrostatic contact potential difference. A feedback loop minimizes the electrostatic interaction force between the tip and sample, adjusting the DC component of a modulated tip voltage. FM-KPFM combined with FM-AFM feedback is used to simultaneously probe the surface potential difference,^11,20^ tip–sample capacitance,^21,22^ mechanical dissipation, and topography of a sample. The implementation is straightforward, but the electrostatic interaction is averaged over a modulation period, and thus, information about the charge dynamics is lost.^20^ The dynamics are crucial to experimentally distinguishing two common but incompatible theoretical explanations of the CISS mechanism in Figure 1.

**Figure 1:**
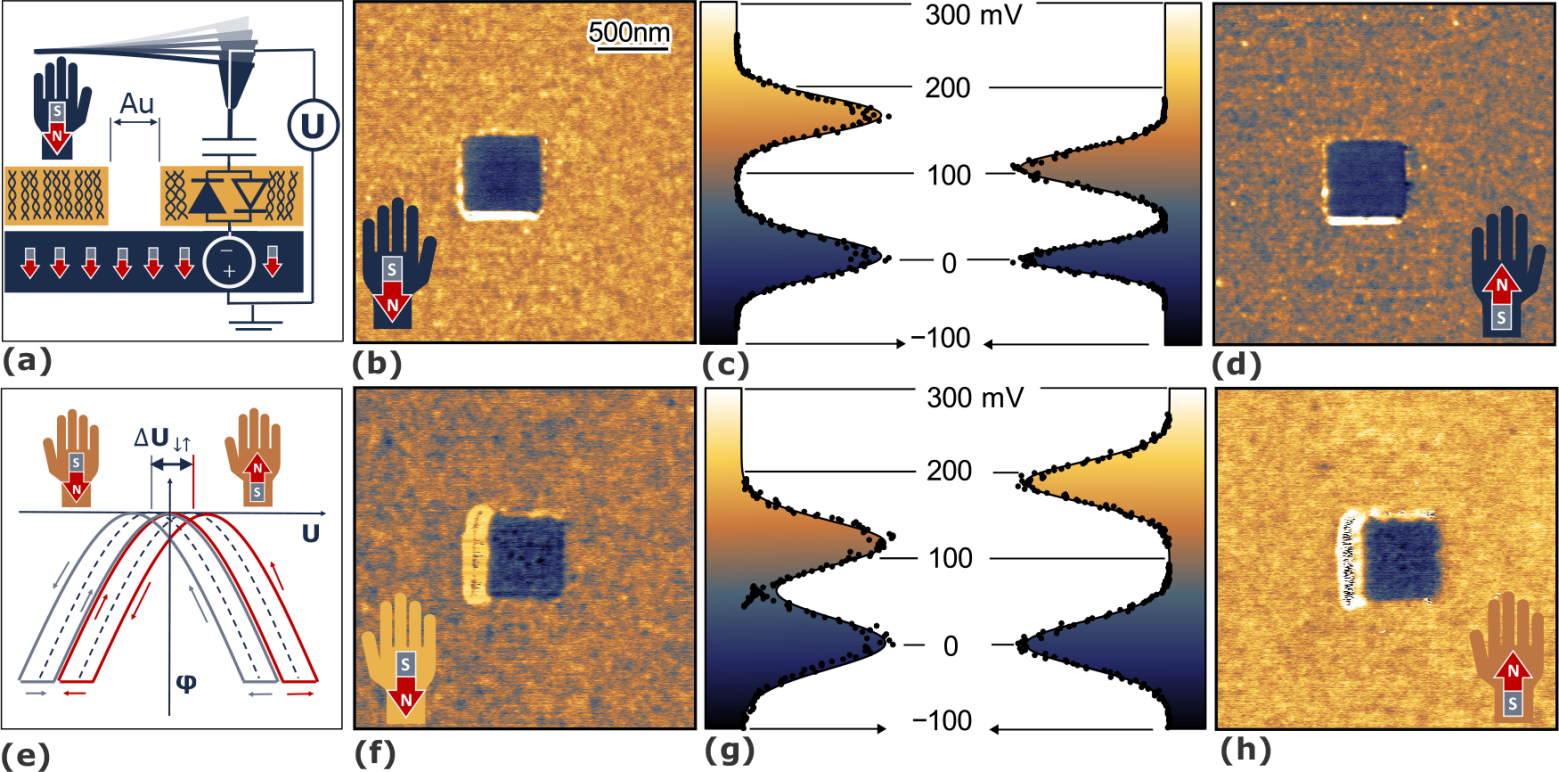
Time-averaged FM-KPFM scans (b–d, f–h) of the contact potential difference *U*_CPD_ showing a Au-reference square surrounded by a peptide monolayer (a) at ω_m_ = 1.4 kHz. The hand^23^ and magnet symbols indicate the configurations (b) L-peptide with magnet pointing to south and (d) L-peptide with magnet pointing to north. (f) D-peptide with magnet pointing south and (h) D-peptide with magnet pointing north. The false color bars (c,g) display the fitted and normalized *U*_CPD_ distributions of b and d and f and g. The potential difference between the Au-reference area (blue peak) and the SAM (yellow peak) is Δ*U* = 165 ± 4 mV (b), 106 ± 4 mV (d), 117 ± 8 mV (f), and 190 ± 8 mV (h). Expected Kelvin parabola hysteresis (e) in south (gray) and north (red) configuration based on the voltage-clamp mechanism of the equivalent circuit in part a.

The first explanation assumes that KPFM perturbed nonequilibrium spin-momentum-locked electron transport^24^ leads to a rectification effect visible in a KPFM-phase hysteresis shown in Figure 1e as solid lines. Two-inverted diodes with different on-set voltages reproduce phenomenological current–voltage characteristics found in CISS transmission experiments^4,25^ (see Figure S8). α-Helix structures have been measured to have nonspin selective diode characteristics^8,26^ and are supported with density functional simulations.^27,28^ In AC-driven configurations,^11,18^ such a current–voltage characteristic could generate a temporal hysteresis and a shift of the time-averaged voltage as shown in Figure 1e. This voltage response to an AC drive is similar to that of a clamping circuit^29^ based on the phenomenological equivalent circuit illustrated in Figure 1a.

The second explanation assumes that the effect is emergent from the interface in absence of charge transfer,^30^ which would show up as the dashed parabola in Figure 1e in KPFM phase. This mechanism is persistent^13^ without global charge transport. For example, spin–orbit interactions, molecular vibrations, electron correlations, and contact with a metallic surface generate an intrinsic spin and charge imbalance in helical molecules in Fransson’s model.^30^ Other models only consider electron reorganization and show similar effects.^31^

With the previous experimental approaches,^11,18^ the time-odd response of the first hypothesis averages out when measuring only the potential difference, impeding the experimental distinction between these two alternative hypotheses. Also, the method of (magnetic) conductive contact AFM^6^ cannot discern and has a few crucial disadvantages compared to noncontact KPFM. In contact AFM, the contact area^32,33^ and thus the specific resistance is difficult to control^34^ and makes quantitative measurements impossible. This is especially relevant in the context of CISS on flexible molecules where deformations in the molecules can strongly influence the result of the measurement.^5^ Nevertheless, routine KPFM is also limited due to cycle-averaging. However, looking at the time response of the surface potential using voltage pumping,^20^ a dynamic distinction is possible. In the following, we showcase this differentiation with time-domain KPFM signal-distance curves. These curves reveal the dynamics and quantum capacitance, a new observable in CISS physics.

The sample consists of 10 nm of nickel (Ni) capped with 5 nm gold (Au) and a self-assembled monolayer (SAM) of left- or right-handed^23^ α-helix forming peptides d- and l-HS–CH_2_–CH_2_–(Ala–Aib)_5_–OH, where Ala refers to alanine and Aib to 2-aminoisobutyric acid. It is a commonly used sequence^35−38^ for CISS experiments. From topography measurements of the SAM, a 46° inclination of peptides can be inferred, which is in agreement with the literature.^25,39^ Experiments were performed under N_2_ to minimize the influence of humidity^40,41^ at a constant temperature of 31.0 ± 0.3 °C to eliminate temperature drift.^38,42^ Positioning the sample on a neodymium disk magnet renders the CISS-induced potential difference measurable. A south configuration designates^43^ the magnetic substrate polarization direction shown in Figure 1a. The respective measurements with north and south orientations are compared to a stable Au-reference since KPFM is sensitive to contaminants. Before the experiment, AFM lithography is used to locally remove the peptide monolayer to obtain a reference (see SI for experimental details). These areas are visible as blue squares in Figure 1.

For the following experiments, open loop time-domain KPFM^44^ is applied. In contrast to the FM-KPFM^19^ configuration in Figure 1b–d,f–h, where a phase-locked loop controls the cantilever phase response, the time-domain KPFM technique directly records the phase-time series. The tip voltage *U* = *U*_DC_ + *U*_AC_ sin ω_m_*t* at ω_m_ = 10 Hz and the tip–sample distance *d*_t_ modulate the signal. Assumed to be at mechanical resonance when the tip and sample are at the same potential, the detected phase is
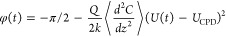
1where *Q* is the quality factor and *k* is the stiffness of the cantilever, ⟨*d*^2^*C*/*dz*^2^⟩ is the cycle averaged capacitance gradient, *U*(*t*) is the tip voltage, and *U*_CPD_ is the contact potential difference (see SI for the detailed derivation). The quadratic dependence on *U* gives rise to the Kelvin parabola shown by dashed lines in Figure 1e. The curvature of the parabola is proportional to the tip–sample capacitance. The tip potential at the maximum of the parabola corresponds to *U*_CPD_.

Figure 2 shows that neither the variation of *U*_AC_ nor the tip–sample distance *d*_t_ influences *U*_CPD_. A greater distance to the sample reduces the capacitance and thus the modulation of the phase. Similarly, a drop of *U*_AC_ leads to a decreased interaction and increased measurement uncertainty. The measured *U*_CPD_ is consistent with the values from the FM-KPFM data of Figure 1, indicating that the effect is independent of the electrical modulation frequency ω_m_. Repeating the experiment but mechanically oscillating the cantilever at the second resonance also confirms the independence for the mechanical resonance ω_0_.

**Figure 2:**
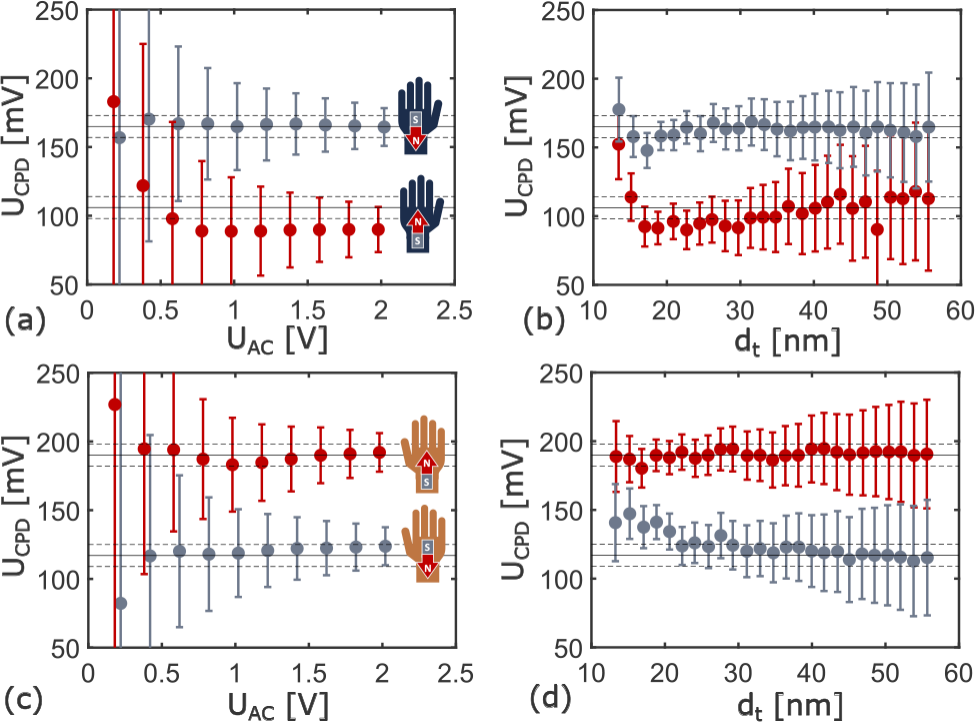
Modulation voltage amplitude *U*_AC_ and the average tip–sample distance *d*_t_ do not affect the surface potential difference *U*_CPD_. Red data corresponds to a north and gray to a south magnetic polarization. Data in a and b are from the l-enantiomer, c and d from the d-enantiomer. The solid black line corresponds to the measured average *U*_CPD_ with FM-AFM/FM-KPFM in the scans of Figure 1 and the dashed to the confidence interval thereof.

Figure 3 shows the phase difference Δφ = φ(*U*_↑_) – φ(*U*_↓_) between the Kelvin parabola of raising *U*_↑_| *∂U*/*∂t* ≥ 0 and falling *U*_↓_| *∂U*/*∂t* < 0 tip-voltage in the four configurations.

**Figure 3:**
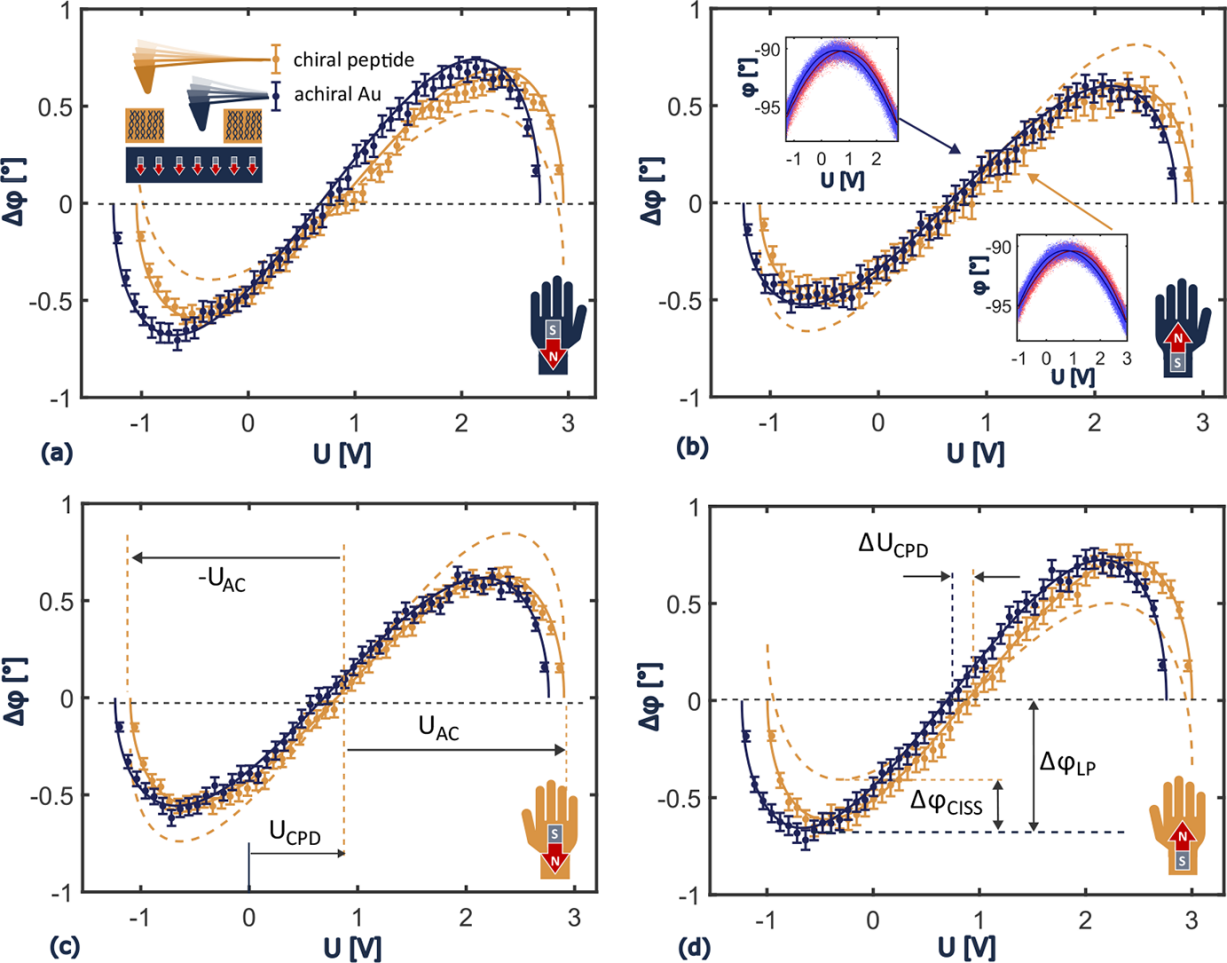
Phase difference Δφ at a calculated distance *d*_t_ = 32 nm in the four different enantiomer and magnetization configurations with one curve each recorded on the Au reference and on peptides. The solid lines correspond to Δφ_LP_, and the dashed lines correspond to Δφ_LP_ + Δφ_CISS_. The insets of b shows the measured time-domain Kelvin parabola where blue data points correspond to *U*_↑_ and red to *U*_↓_.

In case of a voltage-clamp mechanism, the spin-moment locking Δ*U*_*↑↓*_ causes the hysteresial phase difference illustrated in Figure 1e, which is
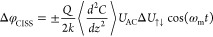
2

There is an additional low pass phase Δφ_LP_ inherent to AFM: If a force on the cantilever changes, this will affect the dynamics instantaneously, but transients add delays to the shift in amplitude and phase.^45^ The transients add phase Δϕ = −arctan(ω_m_/ω_c_) to the Kelvin parabola. ω_c_ = ω_0_/2*Q* ≈ 320 Hz is the cutoff frequency of the cantilever. The resulting intrinsic low pass filter yields the phase difference
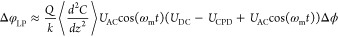
3

The Au reference and the chiral peptide show a similar response; therefore, the asymmetry in the parabola is not a consequence of Δφ_CISS_. The measurements fit Δφ_LP_ exclusively. Figure 4a and c show no Δ*U*_*↑↓*_ at any tip–sample distance *d*_t_, indicating the absence of a global induced spin-momentum locked transport. The simultaneous presence of a potential difference upon toggling magnetization excludes the voltage-clamp mechanism and contrasts transport-based theories.^24,46−48^

**Figure 4:**
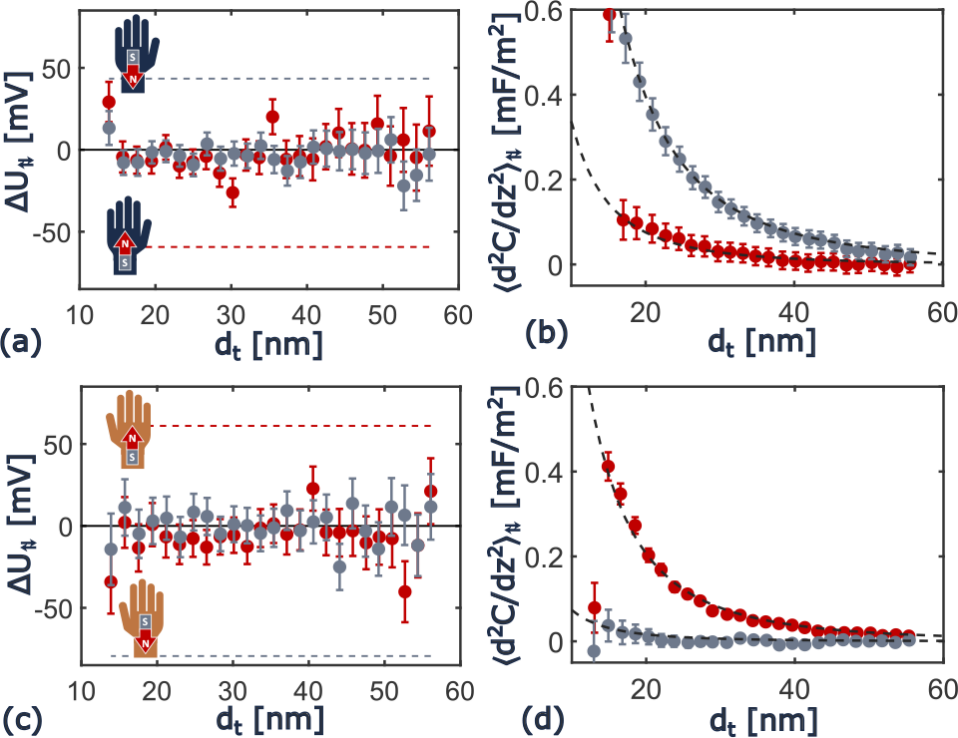
Average tip–sample distance *d*_t_ dependent measurements of the dynamically induced potential shift Δ*U*_*↑↓*_ (a,c) and the capacitance gradient difference ⟨*d*^2^*C*/*dz*^2^⟩_↑↓_ (b,d). Dashed lines correspond to fits of the corresponding models. Red data correspond to a north and gray to a south magnetic polarization. Data in a and b are obtained from the l-enantiomer and c and d from the d-enantiomer.

Contrarily, Figure 4b and d show a substantial variation of the cycle averaged capacitance gradient difference ⟨*d*^2^*C*/*dz*^2^⟩_↑↓_ between the reference and SAM. The difference indicates an additional capacitance specific for a chiral and magnetic configuration. The delocalization of the wave function and its energy quantization adds a quantum contribution to the purely geometric macroscopic capacitance. For a plate capacitor of area *A*, it is^49^
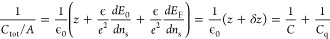
4where *n*_s_ is the density of charges and *e* is the electron charge. The finite occupied density of states inside the SAM changes the effective separation of the capacitor plates by δ*z*.^50^ The second term is associated with the shift of the ground state energy *E*_0_, and the third term is the inverse of the density of states. Those terms contribute to quantum capacitance *C*_q_.

The capacitance^22^ of a tip with effective radius *R* at a distance *z* to a flat metallic sample and quantum distance contribution δ*z* is
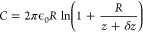
5The fit for the change of *C*_q_ between the reference and chiral monolayer in Figure 4b and d is
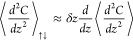
6assuming *δz*/*z* ≪ 1. Fitting ⟨*d*^2^*C*/*dz*^2^⟩ provides *R* = 55 nm and absolute tip–sample distance *d*_t_. Finally, fitting ⟨*d*^2^*C*/*dz*^2^⟩_↑↓_ provides δ*z*. The dashed lines in Figure 4 display the fitted curves obtained from eq 6 using δ*z*_*↓*_ = 1720 ± 60 pm and δ*z*_*↑*_ = 300 ± 80 pm for the l-enantiomer and δ*z*_*↑*_ = 1150 ± 70 pm and δ*z*_*↓*_ = 50 ± 60 pm for the d-enantiomer. The repeatability for distance height curves is within 40 pm, so an arbitrary apparent capacitance due to misalignment is marginal.

Quantum mechanical electrostatics connects all measurements, providing insights into the potential profile at the SAM. Figure 5 shows the effective confinement potential of electrons along the metal–molecule–N_2_–tip junction using measured parameters of the l-peptide in the north (red) and south (gray) configuration. The substrate is grounded. It represents a reservoir for electrons at a chemical potential μ = 0 eV. The density of electrons with a specific spin and their chemical potential inside the ferromagnetic substrate depend on the magnetic polarization.^51^ The molecules are bound to the substrate. They are in chemical and thermal equilibrium with the substrate. Omitting molecular bond details, a triangular quantum well confines the envelope of the multielectron wave function of an isolated molecule in a rough approximation.^52^ The inclined potential represents the polar nature of the chiral molecules.

**Figure 5:**
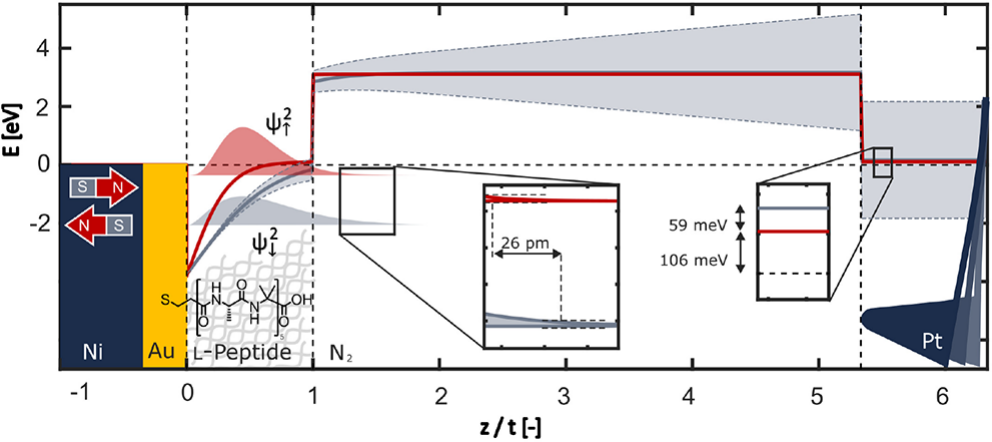
Profile of the effective potentials for electrons. The distance is normalized to the monolayer thickness *t*. The gray dashed outline corresponds to situations where *U*_CPD_ ± 2*V* is applied to the tip, generating a field within the tip–monolayer gap. Gray lines correspond to a high potential, south configuration and red to a low potential, north configuration.

For adsorbed molecules, the confinement persists when the molecules are coupled to an electron reservoir. However, in thermodynamic equilibrium, the number of electrons on the molecule is not identical to the one on isolated molecules.^53^

Although the described problem could be solved self-consistently with numerical methods, the Fang–Howard^49^ variation approach provides valuable conceptual understanding while calculating the envelope wave function. The analytical form of the approach comes at the cost of overestimating the energies. A good approximation for the ground state is the Fang–Howard variational wave function
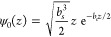
7

The variational parameter *b*_s_ defines the penetration depth of the wave function. The expected charge position is ⟨*z*⟩ = 3/*b*_s_. For simplicity of the model, the electrons on the molecule are treated to be spinless.

The multielectron Hamiltonian can be reduced to a one-dimensional single-particle Hamiltonian confined in a self-consistent Hubbard potential.^49^ Then, a thermal equation of state of the bound surface electrons is derived from the grand canonical ensemble (see SI for details):
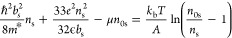
8Since the chiral molecule layer is sensitive to spin polarization^14^
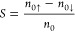
9where the surface charge density *n*_0s_ is considered to depend upon the magnetic polarization *s* = *↑* or *↓* of the Ni layer. The total metal electron density is *n*_0_ = *n*_0*↑*_ + *n*_0*↓*_. Further, from measurements of the quantum capacitance, the effective distance is
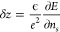
Therefore,
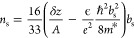
10and *U*_CPD_ is the difference between the tip-potential and the chemical potential, which yields
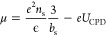
11Plugging eqs 10 and 11 into the thermal state equation (eq 8), one finds *b*_s_. The system is determined when using the measured values for *U*_CPD_ and δ*z* while treating m*, ϵ, *n*_0_, and *S* as constants. The thermal state equation is highly nonlinear in *b*_s_, so a minute change in *b*_s_ impacts the energy, the occupation charge density, and the electronic potential profile.

The literature provides experimentally viable parameters for organic solids using heavy electrons’ effective mass,^54^m* = 25*m*_e_, and a permittivity ϵ = ϵ_r_ϵ_0_ with ϵ_r_ = 2 for peptides.^55^ The Ni–Au stack is estimated to have a spin polarization^51^*S* = 0.33 at an electron density of *n*_0_ = 7.5 nm^–2^. Figure 5 shows the calculated electron density distribution using experimental data supporting predictions of Fransson’s model.^30^ Fransson predicted that CISS induces an electron and spin density reorganization, accompanied by a persistent chemical potential change at the surface without global charge transport. The quantum well in Figure 5 with a magnetic north configuration (red) hosts an average of 2.5 electrons, whereas the south configuration (gray) holds 1.25 electrons.

These different electron densities result from different *b*_s_. The higher the electron density and the higher the *b*_s_, the faster the electric field decays. The fast decay reduces the surface potential, apparent dipole field, and energy. But, a larger *b*_s_ localizes the wave function. The quantum mechanical confinement, but also the charge repulsion, cost energy, reducing the driving force to occupy the quantum well. The spin polarization in the substrate controls the above balance between dipole reduction and confinement. A higher favored spin concentration increases the probability of finding more electrons in the triangular quantum well and reduces the surface potential through screening.

Asymmetric confinement, interaction, and thermodynamic equilibrium are strongly interdependent, can self-enhance each other, and reshape the effective potential.^31^ The system can amplify slight energy differences between spin states to create considerable energy gaps. If one of these three assumptions on which the calculations are based is removed, then the emergent effect is lost.

The potential reconstruction illustrates how bound molecules can drastically change the surface potential and that simple models which only consider added dipole moments^56^ of polar polymers or classic capacitors^57^ fail. However, the presented spinless calculation cannot answer the critical question for CISS of how chirality and spin interactions affect penetration parameter *b*_s_. Combining and refining the model with additional features such as chiral confinement potentials^48,58,59^ or additional interactions^30,60^ will lead to explicitly spin-dependent *b*_s_ but is beyond the scope of this publication.

The electrostatic calculations explain why KPFM does not measurably perturb the system. Data in Figures 2–4 support this conclusion experimentally. Figure 5 illustrates the potential profile in dashed lines for the tip at *U*_CPD_ ± 2 V. It shows that the internal static fields dominate the external dynamic perturbation. In addition, it indicates that tunneling between the tip and sample is extremely unlikely. The following back-of-the-envelope calculation further indicates the minor role of the external KPFM fields: Thermal vibrations of organic compounds have a vibrational amplitude of roughly 5–10% of their binding length. The dipole-induced field oscillates by around the same amount. The intrinsic dipole field is calculated to be 2 V/nm, which agrees with the values of the literature.^57^ The KPFM-induced field is weaker than the dipole fields. However, what seems essential for CISS is the polarization current density, which is proportional to the polarization rate and the molecule polarizability^61^ since the chirality of the electron is proportional to its velocity.^62^ The highest possible frequency component in the present KPFM experiments is mechanical oscillation at 70 kHz, whereas thermal vibrations occur at 10 THz. These orders of magnitude differences in the polarization current further show that externally applied KPFM fields are irrelevant for this experiment.

In scanning tunneling microscopy (STM), the tip–sample distance is much shorter than in AFM. Changes in the local density of states translate to a height difference when scanned at a constant current set point. A recent experiment^7^ finds a 100 pm height difference in a 31-mer polyalanine monolayer. The above wave function converts to a height difference of 26 pm for a 10-mer peptide. Longer molecules lead to higher spin polarization in transport experiments^37,43^ and in the surface potential.^11^ Longer molecules cause a larger electric dipole moment,^31^ which explains the height differences.

Similar reasoning based on wave overlap applies to electron transfer with conductive AFM,^4,25^ molecular junction,^8^ photoemission,^3,4^ and electrochemical transport experiments.^4^ The effect of electron reorganization on electron transfer has been discussed in detail.^31^ The reorganization creates a potential profile with transfer characteristics resembling the diode clamper mechanism.

Time-domain KPFM provides a new method to investigate CISS measuring the potential shift, tracking the slow charging dynamics, and measuring the related change in quantum capacitance simultaneously. Quantum capacitance has previously not been studied experimentally in combination with CISS and provides an observable way to test theoretical models. All measured CISS characteristics are independent of perturbation from KPFM within temporal resolution for α-helical peptides. These experimental findings support that CISS is persistent^13^ and does not require global charge transport. Following theoretical descriptions,^30,31,60^ the measurements show that a persistent charge reorganization^31^ without external charge flow is responsible for the measured capacitance and potential changes. A model based on a triangular quantum well connects all experimental measurements using physically viable parameters and shows the sensitive interplay between the asymmetric confinement potential, the thermodynamic equilibrium of an open system, and the electron–electron interaction to enhance slight spin-sensitive energy differences. Our experiment supports the realization that global charge transport is not required to provoke CISS, implying a paradigm shift in interpreting the effect.

## Supporting Information Available

Detailed description of chemicals and instrumentation, report on peptide synthesis and sample fabrication, plots of reversed-phase high-performance liquid chromatography and high-resolution mass spectrometry, detailed description of KPFM measurements, derivation of KPFM measurands including schemes of equivalent circuits, and the full derivation of the electrostatic model (PDF)
