# Supplementary material for: Detection of a Chirality-Induced Spin Selective Quantum Capacitance in α-Helical Peptides
Source: Nano Lett. 2023 Aug 31;23(17):8280–7. doi: 10.1021/acs.nanolett.3c02483 (PMC10510583; doi:10.1021/acs.nanolett.3c02483)
Supplement: Supplementary file 1 — nl3c02483_si_001.pdf [file nl3c02483_si_001.pdf]

Supporting Information: Detection of a chirality-induced spin selective quantum capacitance in α-helical peptides

Pius Markus Theiler,*,† Christian Ritz,† Raphael Hofmann,‡,¶ and Andreas Stemmer†

†*Nanotechnology Group, ETH Zürich, Säumerstrasse 4, 8803 Rüschlikon, Switzerland*

‡*Laboratory of Organic Chemistry, Department of Chemistry and Applied Biosciences, ETH Zürich, 8093 Zürich, Switzerland.*

¶*Current Address: Laboratory of Bacteriology, The Rockefeller University, New York, NY 10065, USA.*

E-mail: ptheiler@ethz.ch

Chemicals & instrumentation

Peptide synthesis reagents and solvents Acetonitrile (MeCN), *N,N*-dimethylformamide (DMF),methanol (MeOH), diethyl ether (Et_2O), pentane, *N*-methylpyrrolidine (NMP), 4-methylmorphiline (NMM), piperidine, *N,N*-diisopropylethylamine (DIPEA), trifluoroacetic acid (TFA), acetic anhydride, 3-(tritylthio)- propionic acid and triisopropyl silane (TIPS) were purchased from Fisher Scientific (Geel, Belgium), Sigma Aldrich (Buchs, Switzerland), Acros Organics (Geel, Belgium) and TCI Europe (Zwijndrecht, Belgium) and used without further purification (reagent or HPLC grade). Milli-Q water was taken from a Millipore purification system. CH_2Cl_2 was of technical grade and was distilled prior to

use. 2-Chlorotritylchloride polystyrene resin was purchased from Christof Senn Laboratories AG (Dielsdorf, Switzerland). 1-[bis(dimethylamino)methylene]-1H-1,2,3-triazolo[4,5-b]pyridinium 3-oxid hexafluorophosphate (HATU) was purchased from Chem-Impex (Wood Dale, IL, USA). Fmoc-L-Ala-OH, Fmoc-D-Ala-OH and Fmoc-Aib-OH were purchased from Peptides International (Louisville, KY, USA) and Merck (Darmstadt, Germany).

Sample reagents and solvents Ethanol (EtOH, analytical grade), acetone, and iso-propanol (IPA) were purchased from Sigma Aldrich (Buchs, Switzerland). N_2 with a purity >99.999% from Pangas (Zug, Switzerland) was taken from the in-house supply lines.

AFM setup All AFM measurements were performed on an Asylum Cypher ES from Oxford instruments (Santa Barbara, USA) equipped with a ARC 2 controller, a heating-stage ranging from 25 °C to 250 °C, opto-thermal excitation (BlueDrive™), customized tip holder with external electrical tip connection and environmental chamber directly connected to in-house N_2 supply line. The AFM is controlled with the commercial Asylum Cypher Software Package Version 16. Cantilever calibrations are done with the Asylum software function GetReal™. All time-domain and frequency-modulated measurements were demodulated with an external lock-in amplifier HF2LI Zurich Instruments (Zürich, Switzerland) and are controlled with the LabOne™ interface.

Peptide synthesis, purification and characterization

Reversed-phase high-performance liquid chromatography

Reversed-phase high-performance liquid chromatography (RP–HPLC) was performed on a Jasco analytical and preparative instruments with simultaneous monitoring of the eluent at 220 nm, 254 nm and 301 nm at room temperature (rt). The mobile phase was Milli-Q water with 0.1% TFA (solvent A) and HPLC-grade MeCN with 0.1% TFA (solvent B). Analytical

RP–HPLC was performed on a Shiseido C18 (5 μm, 4.6 mm I.D. x 250 mm) column at a flow rate of 1 mL/min using the following method: 10% solvent B for 3 min, followed by a gradient of 10 to 95% solvent B over 14 min and 95% solvent B for 3 min. Preparative RP–HPLC was performed on a YMC C18 (5 μm, 20 mm I.D. x 250 mm) column at a flow rate of 10 mL/min using the following method: 20% solvent B for 5 min, followed by a two-step gradient of 20 to 40% solvent B over 10 min and 40 to 75% solvent B over 20 min. Data was acquired using EZChrom Elite (Version 3.3.2).

High-resolution mass spectrometry (HR–MS)

HR–MS spectra were obtained by the mass spectrometry service of the Laboratory of Organic Chemistry at ETH Zürich on a Bruker Daltonics MaXis ESI–QTOF spectrometer.

Synthesis of peptides

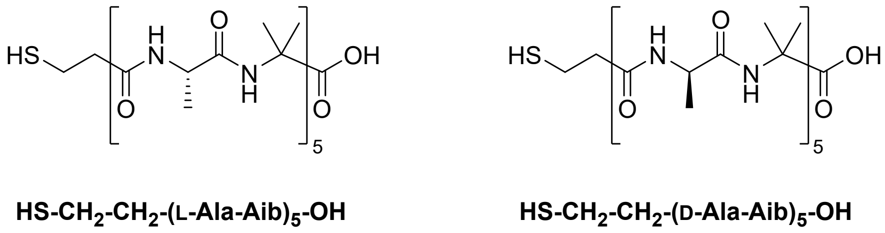

Figure S1: Chemical structure of HS-CH_2-CH_2-(L-Ala-Aib)$_5$-OH and HS-CH_2-CH_2-(D-Ala-Aib)$_5$-OH

Resin loading with Fmoc-Aib-OH 2-Chlorotrityl chloride resin (1.6 mmol/g factory loading) was swollen in CH_2Cl_2/DMF (1:1) for 30 min. To the drained resin was added a solution of Fmoc-Aib-OH (0.25 mmol/g resin, 1 equiv) and NMM (4 equiv) dissolved in a minimal amount of CH_2Cl_2 and a few drops of DMF and the suspension was agitated with N_2 bubbling at rt for 2.5 h. The resin was washed with CH_2Cl_2/DMF (1:1) (6 times), capped with MeOH (5% v/v) and NMM (10% v/v) in DMF with N_2 bubbling for 5 min, and

washed again with CH_2Cl_2/DMF (1:1). The capping step was performed twice. Fmoc-Aib-O-2-chlorotrityl resin was dried under a stream of N_2. Fmoc quantification showed a final amino acid loading of 0.20 mmol/g resin.

Solid-phase peptide synthesis (SPPS) Both peptides were elongated onto Fmoc-Aib-O-2-chlorotrityl resin by automated Fmoc-SPPS at rt using a Multisyntech Syro I parallel synthesizer. For the addition of reagents, Fmoc amino acids and 3-(tritylthio)propionic acid were dissolved in DMF (0.5 M), HATU was dissolved in DMF (0.5 M), and DIPEA was dissolved in NMP (2.0 M). Before syntheses, the resin was swollen in CH_2Cl_2/DMF (1:1) for 30 min. During all reaction steps, the resin was agitated by shaking, and the resin was washed with DMF (6 times) between each step. Fmoc deprotections were performed twice for 10 min with piperidine (20% v/v) in DMF. For peptide couplings, Fmoc amino acid (4.1 equiv relative to synthesis scale), HATU (4.0 equiv) and DIPEA (8.0 equiv) were pre-mixed before addition to the resin. Couplings were performed twice for 45 min. Capping was performed twice for 5 min with acetic anhydride (20% v/v) in DMF mixed with 2 M DIPEA at a ratio of 3:2. Following SPPS, the resin was washed with CH_2Cl_2 and dried under a stream of N_2.

Global deprotection, cleavage from resin, and purification Global deprotection and cleavage from the resin were carried out in TFA/TIPS/H2O (95:2.5:2.5) (1 mL per 100 mg resin) at rt for 1 h and the resin was removed by filtration. Precipitation and trituration of the products with cold Et_2O or pentane was not successful, presumably due to their hydrophobicity. Instead, the entire cleavage solution was concentrated under reduced pressure, the crude residue was dissolved in aqueous MeCN (30% v/v) with TFA (0.1% v/v) and purified by preparative RP–HPLC. Fractions containing pure product were pooled and lyophilized to obtain **HS-CH_2–CH_2–(L-Ala–Aib)$_5$–OH** and **HS–CH_2–CH_2–(D-Ala–Aib)$_5$–OH** as a white solid. The purity and identity of the products were verified by analytical RP–HPLC and HR–MS (results shown below, Fig, S2 - S5).

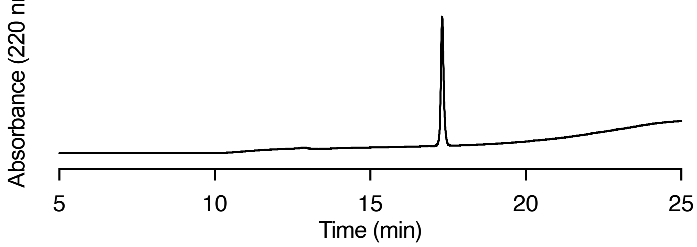

Figure S2: Analytical RP–HPLC of HS–CH_2–CH_2–(L-Ala–Aib)$_5$–OH.

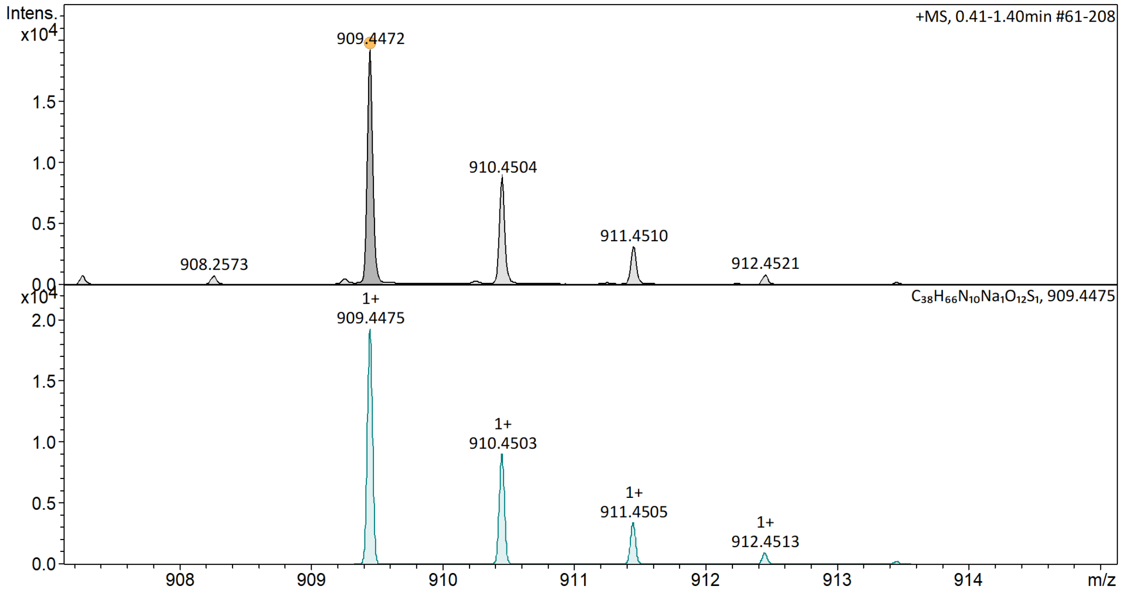

Figure S3: HR–MS (ESI) of HS–CH_2–CH_2–(L-Ala–Aib)$_5$–OH. Obs. 909.4472 (top), calc. for $C_{38}H_{66}N_{10}NaO_{12}S$ [M+Na]+: 909.4475 (bottom).

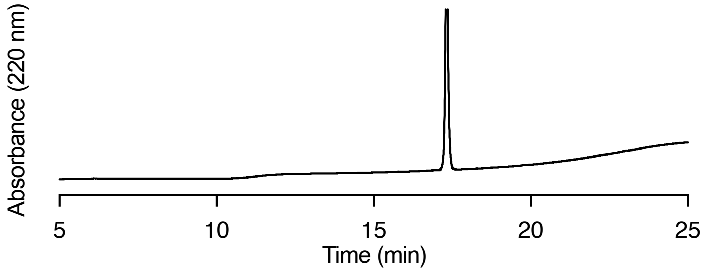

Figure S4: Analytical RP–HPLC of HS–CH_2–CH_2–(D-Ala–Aib)$_5$–OH.

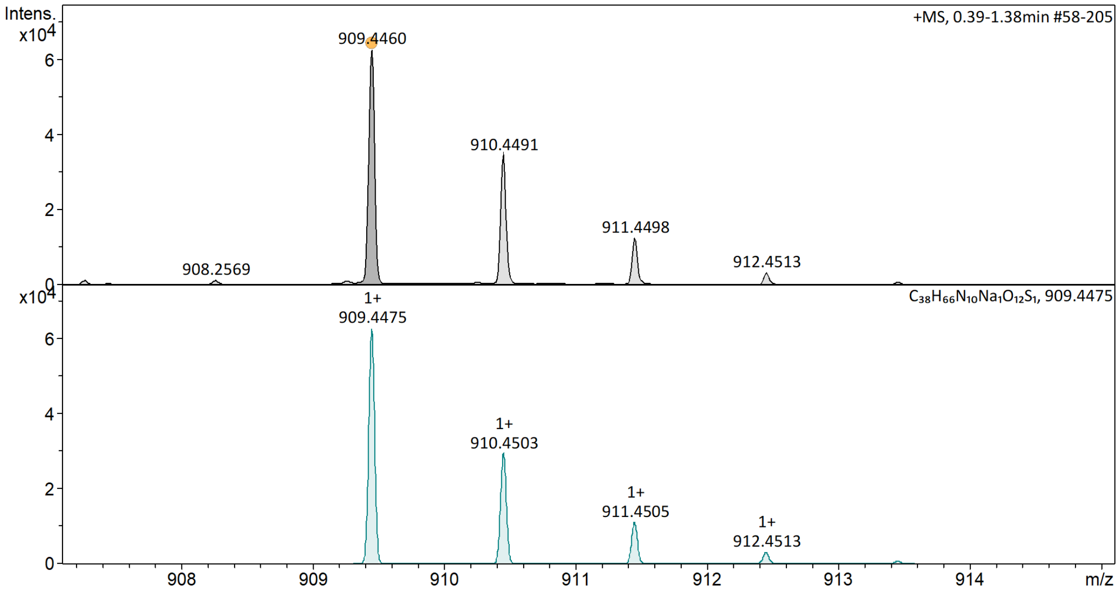

Figure S5: HR–MS (ESI) of HS–CH_2–CH_2–(D-Ala–Aib)$_5$–OH. Obs. 909.4460 (top), calc. for $C_{38}H_{66}N_{10}NaO_{12}S$ [M+Na]+: 909.4475 (bottom).

Sample fabrication

Substrate fabrication Heavily n-doped silicon wafers of 525 μm thickness are cleaned in O_2 plasma for 2min at 500W. A 2 nm thick Ti-adhesion layer, followed by a 10nm Ni and 5 nm of Au, is subsequently e-beam evaporated in an Evatec BAK501 LL at a rate of 0.1 nm/s and a pressure of 1.3 10^{-6} mbar.

Self-assembled monolayer adsorption The substrates are cut into samples of 5x5mm. Each piece is split in half using a diamond pen. The two half-substrates are cleaned with Acetone, IPA, and DI-water in an ultrasonic bath for 10 min each and finally dried with a stream of dry air before immersing the sample into analytical grade EtOH to sonicate for further 10min. In parallel 1 mM L- or D-Peptide in EtOH solvent is prepared and filled into 1 mL plastic Eppendorf cuvettes. Subsequently, one half substrate is transferred into each peptide solution without intermediate drying and incubated for 48 h to form a self-assembled monolayer (SAM). After SAM formation, the substrates are washed three times in analytical grade EtOH by gently shaking for 2min to wash away unbound peptides prior

to drying under a stream of N_2.

Sample mounting for AFM Immediately after drying, the two-half substrates are placed centered onto a neodymium magnet (2 mm thick, 12 mm diameter, S-12-02-N, obtained from www.supermagnete.ch, Switzerland) so that the fracture line is merged. The rough fracture line self-aligns the sample effortlessly within a sub-micron precision several times. In this position, the substrates were fixed with silver paste. It was ensured that the silver paste G 3303B, Leitsilber-deutsches Fabrikat PLANO (Wetzlar,Germany) established direct electrical contact between the metal coatings of the sample and the magnet. The magnetic orientation is checked with a magnetic compass. The magnet is then placed onto an Asylum specimen holder (2 mm thick, 15 mm diameter) and transferred into the environmental chamber of the AFM, where the silver paste is dried under a constant stream of N_2. After 30 min the chamber is reopened to finally position the sample, connect the grounds, to install a tip OMCL-AC160TS Olympus (Tokyo, Japan) for AFM lithography, and check the electrical connection between the sample and ground (the measured resistance is usually below 10 Ω).

AFM lithography Clean metal reference areas are established with AFM lithography on each enantiomer half-sample. For this purpose, the tip is placed within 300 μm of the fracture line so that the tip position can be switched to the opposite side of the fracture (to the opposite enantiomer SAM) without opening the gas chamber. The tip is then fully calibrated (usually k = 22nN/nm, ω_0=296 kHz, Q = 150), and an overview scan in non-contact Amplitude Modulated (AM) AFM mode over 20x20 μm is performed to ensure a flawless monolayer. If successful, the tip is brought into hard contact, and subsequent scans are performed in contact mode at an applied force of 1.3 μN to shear off adsorbed molecules. The 500x500 nm wide area was scanned three times at scanning angles of 0°, 90°, and finally again at 0° to remove all molecules. A control experiment on a sample with no adsorbed monolayer showed no signs of destruction of the scratched gold surface. After the lithography, the result is checked with the same tip in non-contact AM–AFM mode. Finally, the precise

location of the scratched area is documented to locate the position for subsequent KPFM measurements. On each half sample, three reference areas were cleaned. The thickness of the monolayer is $t = 1.07 \pm 0.13$ nm. The length of a 10-mer α-helix is around 1.5 nm, translating to a tilt-angle of 46 $\pm 8°$ (see Fig. S6). Tilting is consistent with angles given in literature.[1] After lithography, the sample is kept under N_2 and only exposed to ambient air when exchanging the AFM tip or flipping the magnetic orientation.

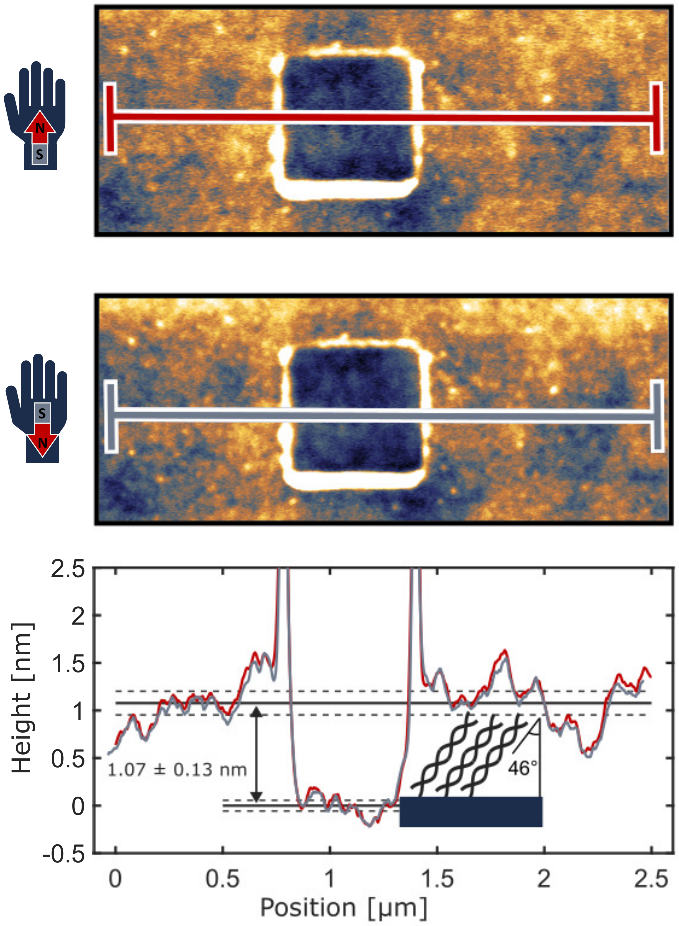

Figure S6: Topography images of the L-peptide-monolayer under north (red) and south (grey) magnetic polarizations cross-section. Solid lines show the mean value, dashed lines the confidence intervals of the height measurements.

Comparison between the blank substrate and scratched substrate The surface potential difference is sensitive to any adsorbed contaminants. To check whether AFM lithography reliably removes all peptides from the surface. The U_{CPD} of a scratched reference

area and the U_{CPD} of the blank sample (only immersed in analytical grade EtOH for 48 h) were compared. No significant difference is found, confirming that the AFM lithography process removed the peptides to create a reliable reference.

AFM measurements

General instrument preparation A fresh Pt coated tip Pt OMCL-AC240TS Olympus (Tokyo, Japan) is mounted, and the magnetization direction is adjusted into the desired direction (see Sample preparation for KPFM). Prior to any measurements, the sample is kept at least 1h under N_2 in the AFM to stabilize the measurement system and minimize humidity. The temperature control is kept active to regulate a temperature of 31 °C. After relocating the reference areas with AM-AFM, the instrument is calibrated. The used tip was calibrated with getReal^{TM} calibration to find $\omega_0 = 75.821$ kHz, $k = 2.32$ nN/nm, $Q = 131$ and the optical lever sensitivity respectively the amplitude $A = 9.5$ nm for the published data set.

Measurement Scheme First, a FM-KPFM FM-AFM scan is performed at each 2.5x2.5 μm reference area. Then a time-domain domain measurement is recorded first on the reference area, then on the SAM at 1 μm away from the reference area. Finally, a second FM-KPFM FM-AFM scan is performed to ensure that the tip did not pick up debris and no damage happened to the sample or the tip during time-domain measurements. This is repeated for all the reference areas prior to switching the magnetic orientation. The magnet is switched at least twice – so that one magnetic configuration has been measured twice to ensure the stability of the system over time. Only data from the identical reference area are compared.

FM-AFM Kelvin probe force microscopy The FM-AFM measurements are based on a single-pass method described elsewhere in detail.[2] In essence, the mechanical oscillation is kept at resonance with a phase-locked loop having a bandwidth of 400 Hz and a range of

5 kHz controlling the topography feedback. The feedback parameters for the phase-locked loop are optimized based on the transfer function of the actual tip. The amplitude of the mechanical oscillation is kept constant at around 9 nm with an amplitude feedback loop adjusting the optothermal drive power. The mechanical oscillation at around ω_0 =75 kHz is modulated with a voltage signal at $\omega_{\mathrm{m}} = 1.4$ kHz and an amplitude of 2 V. The side-bands at $\omega_0 \pm \omega_{\mathrm{m}}$ and $\omega_0 \pm 2\omega_{\mathrm{m}}$ are demodulated at a bandwidth of 100 Hz each. The Kalman filter optimizes the phase of the demodulation signals such that all the signal is in the x-component and adjusts the $U_{\mathrm{DC}} = U_{\mathrm{CPD}}$ to minimize the first side-band. All the feedback loops are run on the lock-in amplifier except for the topography feedback loop. All feedback loops are monitored to operate correctly during scanning. The scans are performed at a speed of 500 nm/s and at a setpoint of 16.5 Hz, which approximately corresponds to an average tip-sample distance of 17 nm. For each scan, the channels for the topography, the frequency-shift (topography error signal), the amplitude drive power, the U_{CPD}, and $\partial^2 C/\partial z^2$ signal are recorded in trace and retrace.

Time-domain Kelvin distance curve measurements The time-domain measurements[3] are recorded open-loop with the lock-in amplifier recording phase, amplitude of the mechanical oscillation, the applied tip voltage and the stage position z at a rate of 3.6 kHz. The lock-in bandwidth is 5 kHz. The mechanical oscillation is modulated with an electrical signal of ω_{m} =10 Hz at an amplitude of 2 V. A static potential U_{DC} is added to the modulation to compensate for contact potential difference. The applied voltage corresponds to the U_{CPD} measured in the FM-KPFM scan. The free, mechanical amplitude is adjusted to 9nm. The z-stage is moved up and down to acquire height-dependent data in a triangular waveform with an amplitude of 60 nm and a velocity of 0.5 nm/s. The return is triggered at a phase shift of 35° – corresponding to a tip-sample spacing of circa 10 nm. After an approach and retract curve are completed, the x-y position is stepped on a 4x4 raster covering an area of 100x100nm to average out local variations of the film.

Data processing FM-KPFM All scan images are processed with the software Gwyddion Version 2.56. All scans are leveled over a vertical line of 125 pixels. The reference strip is completely on the SAM. Distribution statistics are calculated using masks. The masked area excluded the peptide piles at the edges of the reference area. The mean value of the reference area is subtracted to create the basing point.

Data processing time-domain KPFM All time-domain data are processed with the software MATLAB Version R2017b. While importing the raw data, only the position data of the z-sensor is filtered to smooth the data. The data is then cut into the 16 approach and retract curves. Retract and approach curves were treated separately. Generally, data of approach and retract curves overlay, which indicates insignificant drift or hysteresis. Additionally, it shows no mechanical contact (snap-in) with the interface. This is important to ensure the integrity of the SAM. Data with a non-overlapping approach and retract curves are not considered for further analysis (approximately 4% of the data was not useful). The curves are aligned in the z to compensate for topographic roughness using the trigger point as a reference. In the next step, data are binned according to their height and whether the tip voltage was rising or falling. Plotting the applied tip potential versus the phase of the oscillator yields the Kelvin parabola. These raw data were binned over the voltage range. The corresponding bins show an almost perfect normal distribution of the phase values, whereupon the mean value was determined by a normal distribution fit. The error bars show the 95% confidence interval on the difference between the corresponding bins for ascending and descending voltage values.

Capacitance gradient and U_{CPD} values were fitting a parabola with least-squares to the complete data containing falling and raising amplitudes. Errors on the fitted parameters are determined using the covariance matrix and the residual sum of squares. Whenever a result is computed from several parameters, error propagation is applied to determine the total error.

Temperature induced effects

Reversible temperature-induced effects would further support an intrinsic mechanism. Temperature related changes have already been experimentally observed.[4–6] The experimental setup allows for the gathering of temperature-dependent measurements in a limited range between 31°C to 60°C. However, the measurements were not reproducible in the sense that after a heating-cooling cycle, the surface potential changed irreversibly. Heating the sample also leads to a reversible entropic conformation change of the polypeptides,[4] changes in the magnetization(direction) inside the nickel layer by affecting the chemical potential. It also partially demagnetizes the used magnets since the applied temperature is close to the Curie temp of the magnets. The experimental disentanglement of those effects would exceed the scope of this publication.

Derivation of KPFM mesurands

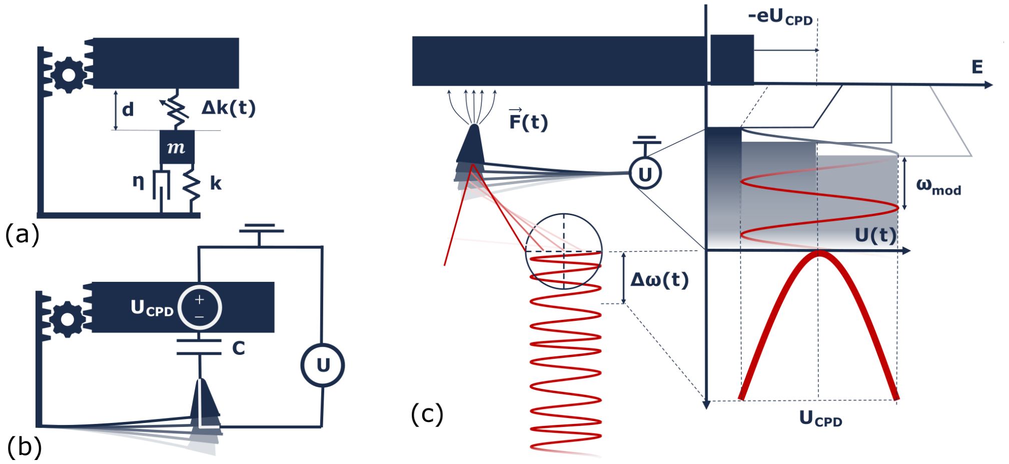

Figure S7: FM-KPFM measurement setup. The measurement system can be described as a force-coupled system consisting of a mechanical, driven oscillator (a) and an electrical equivalent circuit (b). In time-domain KPFM the frequency shift of the mechanical oscillator is recorded while it is mechanically excited (c). An applied voltage between the tip and the sample modulates the electronic states of the sample and generates modulated fields which can be deduced as the phase shift of the mechanical oscillator. The higher the magnitude of the electric field, the lower the oscillator phase. A simple capacitive coupling between the sample and tip is expected for a metallic sample and leads to a Kelvin parabola (c).

Dynamic AFM is sensitive to changes in the oscillator properties determined by the phase, resonance frequency, and amplitude. A change in the spring constant k at fixed excitation frequency ω induces a difference in the phase and amplitude.

Modulating an electrical signal enhances the specificity of the sensor to electrostatic interaction forces between the sample and the tip. The system is a driven mechanical oscillator and an electrical circuit coupled via (electric) force field F_{ts}. Fig. S7 illustrates the equivalent circuit of the electrical system. In the low-frequency range and far away from the surface, the circuit is dominated by parasitic capacitance and resistances and is practically independent of the atomic tip position d. Since the time constant is much shorter than the applied electrical modulation signal, the tip voltage is equivalent to the applied signal.

A harmonic oscillator differential equation models the cantilever dynamics[2,7,8]

$$\ddot{q} + \frac{\omega_0}{Q}\dot{q} + \omega_0^2 q = \omega_0^2 a(t) + \frac{\omega_0^2}{k} F_{\mathrm{ts}}(z_{\mathrm{ts}}, \dot{z}_{\mathrm{ts}}, t), \tag{1}$$

where Q is the quality factor of the cantilever resonance, q the deflection of the cantilever, and $a(t)$ the external excitation signal. The force field F_{ts} is evaluated at the tip-sample distance z_{ts}.

For the analysis shown here, only the non-contact case is considered with $z_{ts} > 0$. Under these circumstances, a periodic excitation $a(t) = a_0 \cos(\omega t + \varphi_{\mathrm{e}})$ produces a periodic response of the resonator which is driven at a constant frequency very close to the resonance, implying $\varphi_{\mathrm{e}} = -\frac{\pi}{2}$. This motivates to write F_{ts} and q as a Fourier series, where

$$F_{\mathrm{ts}}(t) = \frac{f_0}{2} + \sum_{n=1}^{\infty} f_{\mathrm{e},n} \cos(n\omega t) + f_{\mathrm{o},n} \sin(n\omega t) \tag{2}$$

$$q(t) \approx \frac{q_0}{2} + A\cos(\omega t). \tag{3}$$

The strong resonance enhancement of the fundamental harmonic reduces the influence of higher harmonics, such that further analysis can be restricted to the fundamental mode.

Inserting the harmonic ansatz into Eq. (1) and solving for static deflection, amplitude, and frequency leads to

$$q_0 = \frac{f_0}{k} \tag{4}$$

$$A = \frac{Q\omega_0}{\omega}\left(a_0 - \frac{f_{\mathrm{o},1}}{k}\right) \tag{5}$$

$$\omega^2 = \omega_0^2\left(1 - \frac{f_{\mathrm{e},1}}{Ak}\right) \tag{6}$$

with

$$f_0 = \frac{2}{T}\int_0^T F_{\mathrm{ts}}(t)\,dt \tag{7}$$

$$f_{\mathrm{e},1} = \frac{2}{T}\int_0^T F_{\mathrm{ts}}(t)\cos(\omega t)\,dt \tag{8}$$

$$f_{\mathrm{o},1} = \frac{2}{T}\int_0^T F_{\mathrm{ts}}(t)\sin(\omega t)\,dt\,. \tag{9}$$

The time-odd contribution $f_{\mathrm{o},1}$ affects the quality factor of the resonator due to dissipation. Away from the surface, a constant damping can be assumed in good approximation. The time-even contribution $f_{\mathrm{e},1}$ influences the cantilever resonance frequency. The shift in resonance frequency is

$$\Delta\omega = \omega_0\sqrt{1 - \frac{f_{\mathrm{e},1}}{kA}} - \omega_0 \approx -\frac{\omega_0}{2kA}f_{\mathrm{e},1} \tag{10}$$

and translates immediately in a change in phase φ

$$\varphi = \arctan\left(\frac{\omega(\omega_0 + \Delta\omega)}{(\omega^2 - (\omega_0 + \Delta\omega)^2)Q}\right) \tag{11}$$

assuming constant Q and a constant ω not to far away from ω_0 can be linearized to

$$\varphi = \arctan\left(-\frac{\omega_0 + \Delta\omega}{2Q\Delta\omega}\right) \approx -\pi/2 + 2Q\frac{\Delta\omega}{\omega_0} = -\pi/2 + \frac{Q}{kA}f_{\mathrm{e},1} \tag{12}$$

and can be calculated for any force-distance law, provided that the first (even) Fourier

component exists and the harmonic approximation applies.

In KPFM the interaction force is modulated with an electric force.[9] The electrostatic energy[10] stored in the tip-sample capacitance is $E = -\frac{1}{2}C\Delta U^2$, the force is thus

$$F_e(t) = \frac{1}{2}\frac{dC}{dz}U^2 + CU\frac{d\Delta U}{dz} = \frac{1}{2}\frac{dC}{dz}U(t)^2, \tag{13}$$

assuming that the electrical potential difference is independent of the tip-sample distance. This assumption is only valid at tip–sample distances when there is no significant overlap between the quantum mechanical wave functions of the charges of the tip and sample. The potential modulation is of the form $U(t) = U_{\mathrm{DC}} + U_{\mathrm{AC}}\cos(\omega_{\mathrm{m}}t)$ with the modulation frequency $\omega_{\mathrm{m}} \ll \omega_0$. The Fourier coefficients will thus have time-modulated components as well. Using Eq. (8) the component is

$$f_{\mathrm{e},1}(t, z_{ts}) = f_{\mathrm{e},1}(z_t s) + \frac{2}{T}\int_0^T F_{\mathrm{e}}(t)\cos(\omega t)\, dt. \tag{14}$$

In the harmonic approximation, the tip position during a (fast) oscillation is $x = A\cos(\omega t)$ and $dt = -\frac{1}{A\omega\sin(\omega t)}dz = -\frac{1}{\omega\sqrt{A^2-z^2}}dz$, the above integral can be transformed into

$$\frac{2}{T}\int_0^T F_{\mathrm{e}}(t)\cos(\omega t)\, dt = \frac{2}{\pi A}\int_{-A}^{A}\frac{dF_{\mathrm{e}}(z+d)}{dz}\sqrt{A^2-z^2}\, dz = \tag{15}$$

$$\frac{U^2(t)}{2}\frac{2}{\pi A}\int_{-A}^{A}\frac{d^2C(z+d)}{dz^2}\sqrt{A^2-z^2}\, dz = \frac{A}{2}\left\langle\frac{d^2C}{dz^2}\right\rangle U^2(t) \tag{16}$$

by integration by parts to obtain the cycle-averaged force gradient.

Close to the surface $d/R \leq 1$, the electric tip interactions are limited to the appex.[2] The interaction at the tip with an effective radius R is modeled with a sphere at a distance h from a metallic plate with the approximation found in Hudlet *et al.*[11]

$$C(h) = 2\pi\epsilon_0 R\ln\left(1 + \frac{R}{h}\right), \tag{17}$$

and

$$\frac{d^2C}{dh^2} = 2\pi\epsilon_0 \frac{R^2(R+2h)}{z^2(R+h)^2}, \tag{18}$$

and can be used to evaluate the cycle-averaged capacitance. Usual approximations[8] used in contact mechanic AFM are not possible since all length scales are similar. Thus, the gradient is numerically evaluated as a function of minimal tip-sample distance d_t

$$\left\langle \frac{d^2C}{dz^2} \right\rangle = \frac{2}{\pi A^2} \int_{-A}^{A} \frac{d^2C(z+d_t+A)}{dz^2} \sqrt{A^2-z^2}\, dz. \tag{19}$$

Evaluating the above equation and using Eq. 12, the Kelvin parabola for a slow modulation is obtained:

$$\varphi(t) = -\pi/2 - \frac{Q}{2k} \left\langle \frac{d^2C}{dz^2} \right\rangle U^2(t). \tag{20}$$

Modulating with a signal of the form $U(t) = U_{\mathrm{DC}} - U_{\mathrm{CPD}} + U_{\mathrm{AC}} \cos(\omega_{\mathrm{m}})$ yielding a phase signal assuming constant U_{CPD}

$$\varphi(t) = \varphi_0 + \varphi_1 \cos(\omega_{\mathrm{m}} t) + \varphi_2 \cos(2\omega_{\mathrm{m}} t) \tag{21}$$

with

$$\varphi_0 = -\pi/2 - \frac{Q}{2k} \left\langle \frac{d^2C}{dz^2} \right\rangle \left((U_{\mathrm{DC}} - U_{CDP})^2 + \frac{1}{2} U_{AC}^2 \right) \tag{22}$$

$$\varphi_1 = -\frac{Q}{2k} \left\langle \frac{d^2C}{dz^2} (U_{\mathrm{DC}} - U_{CDP}) U_{\mathrm{AC}} \right. \tag{23}$$

$$\varphi_2 = \frac{Q}{4k} \left\langle \frac{d^2C}{dz^2} \right\rangle U_{AC}^2 \tag{24}$$

compensating the average electrostatic force setting $U_{\mathrm{DC}} = U_{\mathrm{CPD}}$, the signal at ω_{m} is minimized and the final signal becomes

$$\varphi(t) = -\pi/2 - \frac{Q}{4k} \left\langle \frac{d^2C}{dz^2} \right\rangle U_{AC}^2 \left(1 - \cos(2\omega_{\mathrm{m}} t + \Delta\phi)\right). \tag{25}$$

If a force on the cantilever changes instantaneously, this will affect the dynamics instantaneously, but transients slow the change in amplitude, phase (or resonance frequency)[7] and add an additional phase delay $\Delta\phi$. Writing the transfer-function for phase detection using the Laplace transform $\mathcal{L}$ and ignoring the electronic phase-detecting system and assuming a small phase change close to resonance $\Delta\phi$ yields

$$G_\varphi(s) = \frac{\mathcal{L}\{\Theta(t)(1-\exp(-\omega_c t))\}}{\mathcal{L}\{(\Theta(t)\}} = \frac{\omega_c}{\omega_c + s} \tag{26}$$

where $\Theta(t)$ is the unit step function. It results in a low pass filter with a cut-off at the critical frequency $\omega_c = \omega_0/2Q \approx 320$ Hz. For slow harmonic phase modulations ($s \ll \omega_\mathrm{m}/\omega_c$) the detected signal is low pass filtered

$$\varphi_d(s) = \frac{\omega_c\omega^2}{s^3 + \omega_c s^2 + \omega^2 s + \omega^2\omega_c} \approx \frac{\omega_c}{\omega_c + s} \tag{27}$$

which introduces a phase delay into the Kelvin parabola of

$$\Delta\phi = -\arctan(\omega_\mathrm{m}/\omega_c) \tag{28}$$

Since $\omega_\mathrm{m}/\omega_c \ll 1$, the phase delays are small, and the observed hysteresis of the phase signal is $\Delta\varphi(t) = \varphi(t) - \varphi(t+\pi/\omega_\mathrm{m})$ due to the phase delays expanding $\varphi(0+\phi) = \varphi(0) + \phi\partial\varphi/\partial\phi|_{\phi=0}$ around null phase delay

$$\Delta\varphi_\mathrm{LP} \approx \frac{Q}{k}\left\langle\frac{d^2C}{dz^2}\right\rangle \Delta\phi U_\mathrm{AC}\sin(\omega_\mathrm{m}t)\Big(U_\mathrm{DC} - U_\mathrm{CPD} + U_\mathrm{AC}\cos(\omega_\mathrm{m}t)\Big). \tag{29}$$

All parameters in this expression are obtained independently from time-resolved measurements and be used as a calibration.

In case of dynamic contact potential difference through spin-moment locking $\Delta U_{\uparrow\downarrow}$, the Kelvin parabola shifts by $\Delta U_{\uparrow\downarrow}$ depending upon the voltage sweep direction (see Fig. S8). Simple electronic circuit analysis yields that an additional phase adds to Eq. 29 due to the

CISS effect

$$\Delta\varphi_{\mathrm{CISS}} = \pm\frac{Q}{2k}\left\langle\frac{d^2C}{dz^2}\right\rangle U_{\mathrm{AC}}\Delta U_{\uparrow\downarrow}\cos(\omega_{\mathrm{m}}t) \tag{30}$$

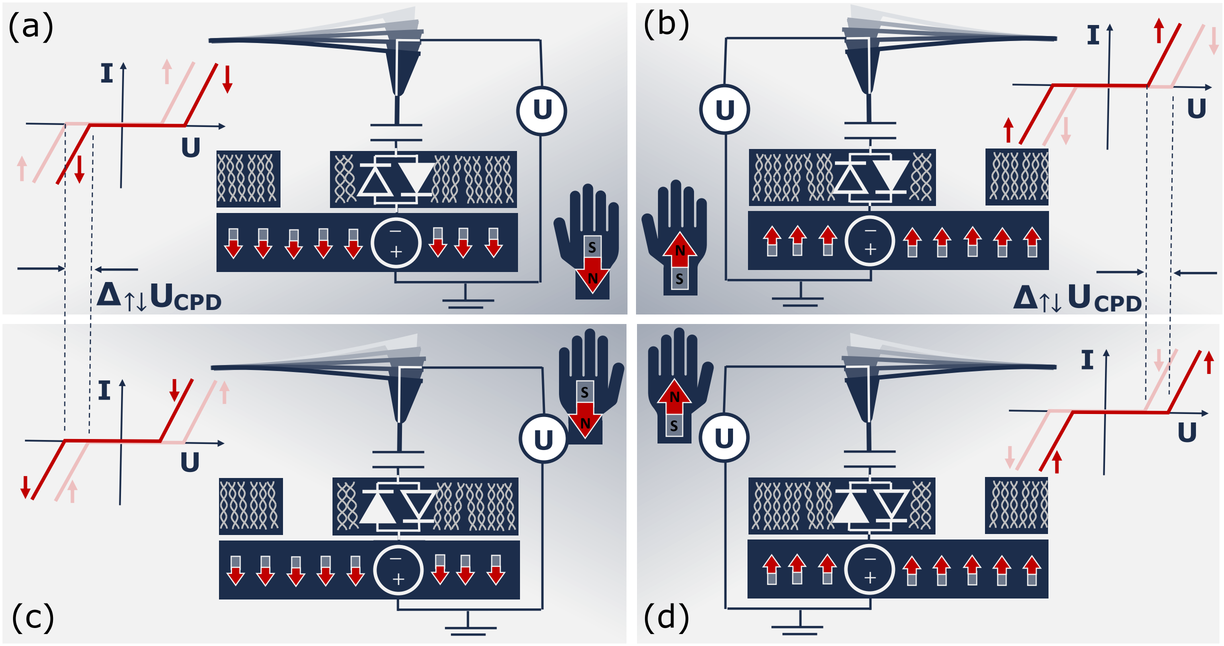

Figure S8: Hypothetical KFPM equivalent circuit for chiral materials. The hand and magnet symbols indicate the configurations (a) L-peptide with magnet pointing to south (b) L-peptide with magnet pointing to north (c) D-peptide with magnet pointing south, and (d) D-peptide with magnet pointing north. Switching the enantiomer leads to an exchange of the on-set voltages of the reversed diodes, whereas switching the magnetic polarisation of the substrate switches the probed I-V characteristic (dark red) and unprobed one (light red).

Derivation of electrostatic potential

The Hamiltonian of the electronic system is

$$H = \sum_i\left[\frac{p_i^2}{2m^*} + \phi(\vec{r}_i)\right] + \frac{1}{2}\sum_{i,j,i\neq j} G(\vec{r}_i,\vec{r}_j) \tag{31}$$

where m^* is the reduced electron mass, $\phi(\vec{r}_i)$ the potential of the i-th electron position and $G(\vec{r}_i, \vec{r}_j)$ the Coulomb interaction inside the molecule electron gas.

We will look at the electron envelope function of a molecule, omitting[12] chemical bonding details assuming a triangular quantum well. The triangular quantum well will account for the polar nature of chiral molecules and the charge reorganization of adsorbed molecules.[13] The molecular layer is assumed to be homogeneous in in-plane directions.

Assuming that the wave function does not penetrate the metal layer simplifies calculations; therefore, the potential is infinite in the left half space. It is thus sufficient to consider $z > 0$. The electron-electron interactions are treated within the self-consistent Hartree approximation. The approach is inspired by the treatment of a 2D electron gas[14] and is reduced to 1D. The single particle-envelope wave function ψ_n fulfills the Hartree equation

$$\left[-\frac{\hbar^2}{2m^*}\frac{\partial^2}{\partial z^2} + V_H(z)\right]\psi_n(z) = E_n\psi_n(z) \tag{32}$$

A good approximation for the ground state of the Fang-Howard variational wave function is

$$\psi_0(z) = \sqrt{\frac{b_s^3}{2}} z e^{-b_s z/2} \tag{33}$$

The variational parameter b_s defines the penetration depth of the wavefunction such that the system is in thermodynamic equilibrium with the metal. It can also be expressed in terms of the expected charge position $\langle z \rangle = 3/b_s$. Within the approach, an upper bound for the system's ground energy per unit area is

$$\langle E \rangle = \langle \hat{T} \rangle + \frac{1}{2}\langle \hat{V}_H \rangle. \tag{34}$$

The energy is composed of a single particle kinetic term $\langle \hat{T} \rangle$ and an electron-electron interaction term $\langle \hat{V}_H \rangle$

$$\langle\hat{T}\rangle = \frac{\hbar^2 b^2}{8m^*} n_s \tag{35}$$

.

In order to find the Hartree interaction energy, the electron density distribution is

$$\rho(z) = -|e| n_s |\psi_0|^2, \tag{36}$$

and applying Poisson's equation

$$\frac{\partial V_H}{\partial z} = \frac{|e|\rho(z)}{\epsilon}, \tag{37}$$

imposing that the field at the interface corresponds to the field of the dipole moment of the molecule

$$\frac{\partial V_H(0)}{\partial z} = \frac{e^2 n_s}{\epsilon} = \vec{E}_{int} \tag{38}$$

and setting $V_H(0) = 0$ with no loss of generality. Solving the equation leads to

$$V_H(z) = \frac{e^2 n_s}{\epsilon} \frac{1}{2b_s} \left[6 - ((b_s z)^2 + 4 b_s z + 6) e^{-b_s z}\right], \tag{39}$$

and after integrating the Coulomb interaction energy is

$$\langle\hat{V}_H\rangle = \frac{33 e^2 n_s^2}{16 \epsilon b_s} \tag{40}$$

where n_s corresponds to the area-related electron density trapped in the potential. The expected energy per area of the bound electrons is

$$\langle E\rangle = \frac{\hbar^2 b^2}{8m^*} n_s + \frac{33 e^2 n_s^2}{32 \epsilon b_s}. \tag{41}$$

The system is in thermal equilibrium with the substrate at a chemical potential μ and can exchange electrons with the gold-nickel stack. The grand-canonical partition function of

the system is

$$\mathcal{Z} = 1 + e^{\frac{\mu n_{0s} A - AE}{k_b T}} \tag{42}$$

where n_{0s} is the area-related electron density of the metal and A the base area of a molecule. With the fugacity $z = e^{\mu/k_b T}$, the electron density n_s calculating the electron number expectation

$$n_s = \langle N \rangle = z \frac{\partial}{\partial z} \ln \mathcal{Z} = \frac{n_{0s}}{1 + e^{\frac{AE - \mu n_{0s} A}{k_b T}}} \tag{43}$$

is obtained. Rearranging and the usage of Eq. 41 yields a thermal equation of state for the bound electrons:

$$\frac{\hbar^2 b^2}{8m^*} n_s + \frac{33 e^2 n_s^2}{32 \epsilon b_s} - \mu n_{0s} = \frac{k_b T}{A} \ln \left(\frac{n_{0s}}{n_s} - 1 \right). \tag{44}$$

Since the chiral molecule layer is sensitive to spin polarization S, n_{0s} is considered to depend upon the magnetic polarization $s =\uparrow$ or $\downarrow$ of the nickel layer

$$S = \frac{n_{0\uparrow} - n_{0\downarrow}}{n_0} \tag{45}$$

where the total metal electron density is $n_0 = n_{0\uparrow} + n_{0\downarrow}$.

Further, from measurements of the quantum capacitance, the effective distance $\delta z = \frac{\epsilon}{e^2} \frac{\partial E}{\partial n_s}$ because the Fermi energy is kept constant due to grounding and becomes after solving for n_s

$$n_s = \frac{16}{33} \left(\frac{\delta z}{A} - \frac{\epsilon}{e^2} \frac{\hbar^2 b_s^2}{8m} \right) b_s, \tag{46}$$

and U_{CPD} is obtained as the difference of the limit $z \to \infty$ of the Hubbard potential $V_H(z)$ and the chemical potential, so

$$\mu = \frac{e^2 n_s}{\epsilon} \frac{3}{b_s} - eU_{\text{CPD}} \tag{47}$$

Plugging 46 and 47 into the thermal state equation (Eq.44), b_s determines the system completely using the measured values for U_{CPD} and δz while treating m^*, ϵ, n_0 and S as parameters. The thermal state equation is highly non-linear in b_s, so a minute change in b_s

impacts on the energy, the occupation charge density, and the potential profile.

References

(1) Ha, N. T.; Sharma, A.; Slawig, D.; Yochelis, S.; Paltiel, Y.; Zahn, D. R.; Salvan, G.; Tegenkamp, C. Charge-ordered α-helical polypeptide monolayers on Au(111). *Journal of Physical Chemistry C* **2020**, *124*, 5734–5739.

(2) Wagner, T.; Beyer, H.; Reissner, P.; Mensch, P.; Riel, H.; Gotsmann, B.; Stemmer, A. Kelvin probe force microscopy for local characterisation of active nanoelectronic devices. *Beilstein Journal of Nanotechnology* **2015**, *6*, 2193–2206.

(3) Ritz, C.; Wagner, T.; Stemmer, A. Measurement of electrostatic tip – sample interactions by time-domain Kelvin probe force microscopy. *Beilstein Journal of Nanotechnology* **2020**, *11*, 911–921.

(4) Eckshtain-Levi, M.; Capua, E.; Refaely-Abramson, S.; Sarkar, S.; Gavrilov, Y.; Mathew, S. P.; Paltiel, Y.; Levy, Y.; Kronik, L.; Naaman, R. Cold denaturation induces inversion of dipole and spin transfer in chiral peptide monolayers. *Nature Communications* **2016**, *7*, 1–9.

(5) Mondal, A. K.; Brown, N.; Mishra, S.; Makam, P.; Wing, D.; Gilead, S.; Wiesenfeld, Y.; Leitus, G.; Shimon, L. J.; Carmieli, R.; *et al.*, Long-range spin-selective transport in chiral metal-organic crystals with temperature-activated magnetization. *ACS Nano* **2020**, *14*, 16624–16633.

(6) Das, T. K.; Tassinari, F.; Naaman, R.; Fransson, J. Temperature-dependent chiral-induced spin selectivity effect: experiments and theory. *Journal of Physical Chemistry C* **2022**, *126*, 3257–3264.

(7) Wagner, T. Steady-state and transient behavior in dynamic atomic force microscopy. *Journal of Applied Physics* **2019**, *125*, 044301.

(8) Theiler, P. M.; Ritz, C.; Stemmer, A. Shortcomings of the Derjaguin – Muller – Toporov model in dynamic atomic force microscopy. *Journal of Applied Physics* **2021**, *130*, 244304.

(9) Nonenmacher, M.; O'Boyle, M.; Wickramasinghe, H. Kelvin probe force microscopy. *Applied Physics Letters* **1991**, *58*, 2921–2923.

(10) Kantorovich, L. N.; Livshits, A. I.; Stoneham, M. Electrostatic energy calculation for the interpretation of scanning probe microscopy experiments. *Journal of Physics Condensed Matter* **2000**, *12*, 795–814.

(11) Hudlet, S.; Jean, M. S.; Guthmann, C.; Berger, J. Evaluation of the capacitive force between an atomic force microscopy tip and a metallic surface. *The European Physical journal B* **1998**, *2*, 5–10.

(12) Rissner, F.; Egger, D. A.; Natan, A.; Körzdörfer, T.; Kümmel, S.; Kronik, L.; Zojer, E. Collectively induced quantum-confined stark effect in monolayers of molecules consisting of polar repeating units. *Journal of the American Chemical Society* **2011**, *133*, 18634–18645.

(13) Naaman, R.; Waldeck, D. H.; Fransson, J. New perspective on electron transfer through molecules. *Journal of Physical Chemistry Letters* **2022**, *13*, 11753–11759.

(14) Ihn, T. *Semiconductor nanostructures*; Oxford University Press: New York, 2015; pp 115–121.
